# Managing disorders of consciousness: the role of electroencephalography

**DOI:** 10.1007/s00415-020-10095-z

**Published:** 2020-09-11

**Authors:** Yang Bai, Yajun Lin, Ulf Ziemann

**Affiliations:** 1 International Vegetative State and Consciousness Science Institute, Hangzhou Normal University, Hangzhou, China; 2 Department of Neurology and Stroke, University of Tübingen, Hoppe‑Seyler‑Str. 3, 72076 Tübingen, Germany; 3 Hertie Institute for Clinical Brain Research, University of Tübingen, 72076 Tübingen, Germany

**Keywords:** Disorders of consciousness, Electroencephalography, Minimally conscious state, Vegetative state, Unresponsive wakefulness state, Event-related potential, TMS-EEG

## Abstract

Disorders of consciousness (DOC) are an important but still underexplored entity in neurology. Novel electroencephalography (EEG) measures are currently being employed for improving diagnostic classification, estimating prognosis and supporting medicolegal decision-making in DOC patients. However, complex recording protocols, a confusing variety of EEG measures, and complicated analysis algorithms create roadblocks against broad application. We conducted a systematic review based on English-language studies in PubMed, Medline and Web of Science databases. The review structures the available knowledge based on EEG measures and analysis principles, and aims at promoting its translation into clinical management of DOC patients.

## Introduction

A disorder of consciousness (DOC) is an altered consciousness state generally caused by injury or dysfunction of the neural systems regulating arousal and awareness [1, 2]. When patients fall into coma after brain injury, they show an absence both of arousal (i.e., eye opening even when stimulated) and awareness (unaware of themselves and the environment) [3]. After the transition from coma, some patients remain unresponsive to external stimulation (or only show simple reflex movements that are uncorrelated with command), and they will be diagnosed as being in a vegetative state/unresponsive wakefulness syndrome (VS/UWS) [4]. Moreover, patients who show fluctuating but definite behavioral evidence of self or environmental awareness can be diagnosed as in minimally conscious state (MCS) [5]. The current ‘gold standard’ for establishing a diagnosis of VS/UWS or MCS is still behavioral scales like the JFK Coma Recovery Scale—Revised (CRS-R) [6]. However, it is well accepted that this scale has significant limitations [7]. Due to fluctuations of consciousness and fatiguability of the patients and subjective interpretation, assessments based exclusively on behavioral scales can lead to misdiagnosis rate as high as 40% [8].

Clinicians and neuroscientists now increasingly often seek to obtain information of brain function from neuroimaging or electrophysiological measurements for a more precise assessment of DOC patients. Compared with neuroimaging techniques, electroencephalogram (EEG) recordings are a more widely applicable, less expensive, and more practical technique for application in DOC patients. Many EEG studies in DOC patients have been conducted already and provided valuable information for clinicians to improve the diagnosis, monitoring and prognosis of these patients. However, the EEG paradigms, analysis algorithms and feature extraction are complex. Many EEG-based characteristics have been proposed to describe the pathophysiology underlying DOCs. They have enriched clinical methods for DOC assessment, but at the same time, they have confused clinicians when it comes to selecting the appropriate ones in practice. The purpose of this systematic review is to classify the EEG literature in DOC patients according to protocols, analysis methods and clinical utility.

## Literature sources

English-language articles were searched in PubMed, Medline and Web of Science databases published up to 15 May 2020. Key search terms were ‘disorders of consciousness; vegetative state; unresponsive wakefulness syndrome and minimally conscious state’ combined with ‘electroencephalogram; event-related potentials; brain–computer interfaces; sleep; EEG reactivity; TMS-EEG’. A total of 753 articles were identified. Title and abstract of the retrieved articles were then screened for the following inclusion criteria: (1) original peer-reviewed article; (2) subjects diagnosed as DOC; (3) application of EEG measurement and analysis. Exclusion criteria were: (1) review and guideline articles, and conference proceedings and abstracts; (2) studies on children, acute comatose state, or without explicit EEG results. The retained 119 articles provide the basis for this review and were classified according to EEG measures, paradigms, analysis methods and clinical utility.

## Results

**Fig. 1:**
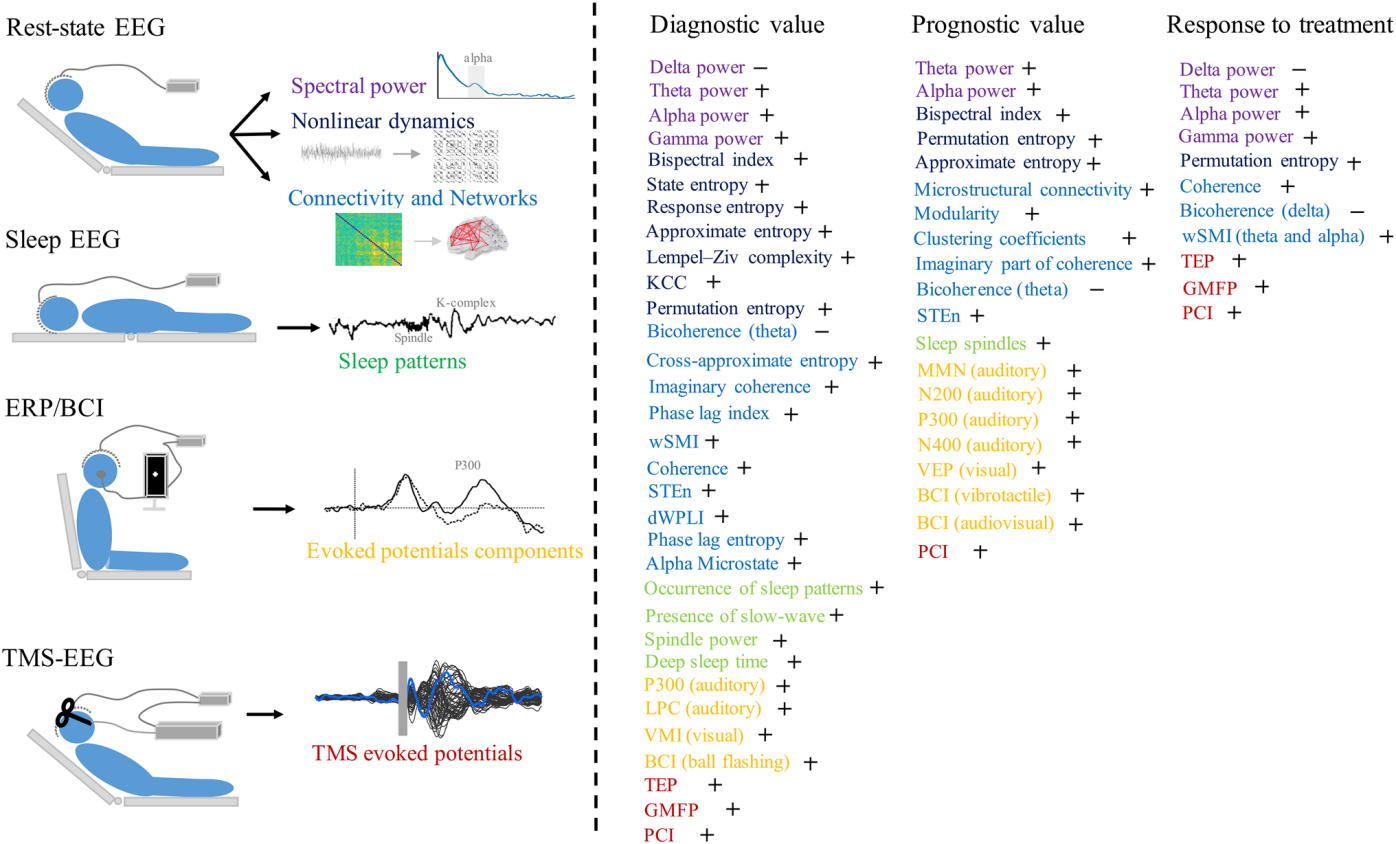
Main EEG measures obtained in patients with disorders of consciousness: rest-state EEG, sleep EEG, event-related potentials (ERP), brain-computer interface (BCI) and transcranial magnetic stimulation–electroencephalography (TMS-EEG). These measures provide various EEG characteristics based on different information extraction algorithms. The right panel summarizes the main EEG characteristics with respect to their potential values in classifying minimally conscious state (MCS) and vegetative state/unresponsive wakefulness syndrome (VS/UWS) (diagnostic value), outcomes prediction (prognostic value) and treatment monitoring (response to treatment). The colors identify EEG measures belonging to the main EEG techniques as indicated by the same colors in the left panel. ± in the ‘diagnostic value’ column indicate that EEG measures are larger (+)/smaller (–) in MCS than in VS/UWS. ± in the ‘prognostic value’ column indicate that larger EEG measures correspond to better (+)/worse (–) outcome. Finally, ± in the ‘response to treatment’ column means that EEG measures increased/decreased during treatment. *KCC* Kolmogorov–Chaitin complexity, *wSMI* weighted symbolic mutual information, *dwPLI* debiased weighted phase lag index, *STEn* symbolic transfer entropy, *LPC* late positive components, *VMI* visuomotor integration, *VEP* visual evoked potentials, *MMN* mismatch negativity, *TEP* TMS evoked potentials, *GMFP* global mean field power, *PCI* perturbational complexity index

EEG measurements include spontaneous (resting-state EEG: Tables 1, 2, 3, and sleep EEG: Table 4) and evoked EEG (event-related potentials, brain-computer interfaces: Table 5, EEG-reactivity and transcranial magnetic stimulation evoked EEG responses: Table 6). The different paradigms, EEG characteristics and their potential clinical utility are summarized in Fig. 1.

**Table 1:** EEG spectral power measurements in disorders of consciousness

Clinical application	Literature	Frequency bands	Subjects (numbers)	Main findings
Diagnosis	Coleman et al. [27]	Ratio between lower frequencies (delta and theta) and higher frequencies (alpha and beta)	MCS 4 VS/UWS 6	Correlation between EEG power ratio index and regional glucose metabolism was preserved in all MCS, but was absent in all VS/UWS VS/UWS showed a significantly higher EEG power ratio index in comparison with the MCS
	Leon-Carrion et al. [17]	Delta and theta	SND 9 MCS 7	Posterior sources of delta and theta frequencies had higher amplitude in MCS patients than in SND patients
	Babiloni et al. [117]	Delta and alpha	HC 13 LIS 13	Power of alpha2 (individual alpha frequency -2 to individual alpha frequency) and alpha3 (individual alpha frequency to individual alpha frequency + 2) in all regions was lower in patients with LIS compared to HC The power of delta in central, parietal, occipital and temporal regions was higher in patients with LIS compared to HC
	Lehembre et al. [20]	Delta and alpha	MCS 18 VS/UWS 10	VS/UWS showed increased delta power but decreased alpha power compared with MCS
	Lechinger et al. [12]	Delta, theta, alpha and ratio	HC 14 MCS 9 VS 8	VS/UWS showed higher delta and theta than HC Alpha activity was strongly decreased in both MCS and VS/UWS as compared to HC Positive correlation between CRS-R score and the power ratio of above 8 Hz to below 8 Hz
	Sitt et al. [13]	Delta and alpha	HC 14 CS 24 MCS 68 VS/UWS 75	Increased normalized delta power separated VS/UWS from MCS and from CS patients, whereas the converse occurred in higher frequency bands, with decreased normalized alpha power segregating VS/UWS from MCS and from CS patients
	Chennu et al. [16]	Delta and alpha	HC 26 MCS 19 VS/UWS 13	80% of overall spectral power in VS/UWS was concentrated within the delta band
	Rossi Sebastiano et al. [11]	Delta and alpha	MCS 57 VS/UWS 85	VS/UWS had a significantly higher relative delta power in fronto-central and parieto-occipital than MCS Significant correlations between CRS scores and relative delta power and relative alpha power in the fronto-central, parieto-occipital, and midline regions
	Piarulli et al. [14]	Delta, theta and alpha	MCS 6 VS/UWS 6	MCS patients had higher theta and alpha and lower delta power than VS/UWS
	Naro et al. [18]	Delta, theta, alpha, beta, and gamma	HC 10 MCS 7 VS/UWS 6	Alpha significantly correlated with the consciousness level MCS showed higher alpha, gamma and theta, as well as lower delta than VS/UWS
	Naro et al. [25]	Theta and gamma	HC 10 MCS 10 VS/UWS 10	DOC patients showed higher theta and lower gamma power than HC
	Stefan et al. [19]	Delta and alpha	MCS 11 VS/UWS 51	Alpha power was greater for MCS patients than VS/UWS patients, and conversely for delta power
	Naro et al. [15]	Delta and alpha	MCS 15 VS/UWS 17	Nearly 80% of the overall spectral power was concentrated within the delta band in patients with VS/UWS MCS patients showed an alpha power twice the patients with VS/UWS Delta power decreased and the alpha power increased with increase of CRS-R score

Clinical application	Literature	Frequency bands	Follow-up times	Subjects (numbers)	Main findings
Prognosis	Babiloni et al. [22]	Alpha	3 months	HC 30 VS/UWS 50	The higher of alpha power, the higher the chance to recover consciousness
	Sitt et al. [13]	Theta	< 42 days	HC 14 CS 24 MCS 68 VS/UWS 75	Patients recovering from VS/UWS tended to exhibit a higher normalized theta power, relative to non-recovering patients
	Stefan et al. [19]	Delta and alpha	589 ± 1125 days	MCS 11 VS/UWS 51	Alpha and delta performed better at discriminating outcome than indexing consciousness

Clinical application	Literature	Frequency band	Treatment	Subjects (numbers)	Main findings
Treatment	Williams et al. [48]	∼6–10 Hz activity	Zolpidem	Three diverse patients with known zolpidem responses	Behavioral activation with zolpidem reliably attenuated ~ 6-10 Hz power and was associated with an increase in power at ~ 15-30 Hz
	Naro et al. [25]	Theta and gamma	otDCS	HC 10 MCS 10 VS/UWS 10	otDCS increased theta and gamma power in healthy and MCS but not in VS/UWS
	Naro et al. [24]	Gamma	tACS	HC 15 MCS 12 VS/UWS 14	tACS entrained theta and gamma oscillations and strengthened the connectivity patterns within frontoparietal networks in all HC and MCS, and some VS/UWS
	Bai et al. [26]	Delta and gamma	SCS	MCS 11	Relative power of delta significantly decreased, while gamma increased after one session of SCS
	Hermann et al. [23]	Full band	tDCS	EMCS 4 MCS 32 VS/UWS 24	Responders showed a significant increase in normalized theta power with a maximal effect over the parietal cortices and an increase of both raw and normalized alpha power compared to non-responders

*HC* healthy control, *DOC* disorders of consciousness, *EMCS* emerged form minimally conscious state, *MCS* minimally conscious state, *VS/UWS* vegetative state/unresponsive wakefulness state, *LIS* lock-in syndrome, *CRS-R* JFK Coma Recovery Scale-Revised, *SND* severe neurocognitive disorders, *CS* conscious patients, *tDCS* transcranial direct current stimulation, *otDCS* oscillatory transcranial direct current stimulation, *tACS* transcranial alternating current stimulation, *SCS*  spinal cord stimulation

**Table 2:** Nonlinear EEG measurements in disorders of consciousness patients

Clinical application	Literature	Non-linear measurements	Subjects (numbers)	Main findings
Diagnosis	Gosseries et al. [32]	State entropy and response entropy	HC 16 MCS 26 VS/UWS 24 Coma 6	EEG entropy correlated with CRS-R total scores Mean EEG entropy values were higher in MCS than in VS/UWS
	Sara et al. [29]	ApEn	HC 40 VS/UWS 38	Mean ApEn values were lower in patients than in HC
	Wu et al. [28]	LZC, ApEn	HC 30 MCS 16 VS/UWS 21	VS/UWS had the lowest LZC and ApEn, followed by MCS, and HC had the highest
	Sitt et al. [13]	PE, KCC	HC 14 CS 24 MCS 68 VS/UWS 75	PE-based measures were particularly efficient in the theta frequency range, discriminating VS/UWS from other groups KCC significantly discriminated VS/UWS from MCS, particularly for electrodes over parietal region
	Piarulli et al. [14]	Spectral entropy	MCS 6 VS/UWS 6	MCS showed higher spectral entropy mean value and higher time variability MCS were characterized by spectral entropy fluctuations with periodicities at 70 min, the periodicities closely resemble those described in awake HC
	Thul et al. [40]	PE	HC 24 MCS 7 VS/UWS 8	VS/UWS showed considerably reduced PE compared to MCS Overall differences of PE were significant between HC and MCS, and between HC and VS/UWS
	Stefan et al. [19]	ApEn and PE	MCS 11 VS/UWS 51	ApEn in all frequency ranges was higher in MCS than VS/UWS PE in the alpha range was significantly higher in MCS than VS/UWS
	Engemann et al. [30]	PE, KCC and Spectral entropy	MCS 179 VS/UWS 148	PE was one of the top ranked among > 20 potential biomarkers for classification of of DOC patients

Clinical application	Literature	Non-linear measurements	Subjects (numbers)	Follow-up times	Main findings
Prognosis	Sara et al. [29]	ApEn	HC 40 VS/UWS 38	6 months	Patients with the lowest ApEn values either died (*n* = 14) or remained in a VS/UWS (*n* = 12), whereas patients with the highest ApEn values became MCS (*n* = 5) or showed partial (*n* = 4) or full recovery (*n* = 3)
	Wu et al. [28]	LZC, ApEn	CS 30 MCS 16 VS/UWS 21	6 months	LZC and ApEn under painful stimulation increased more significantly in patients who recovered than in patients without recovery
	Stefan et al. [19]	ApEn, PE	MCS 11 VS/UWS 51	589 ± 1125 days	PE in the delta, theta bands predicted outcome better than ApEn

Clinical application	Literature	Non-linear measurements	Subjects (numbers)	Treatments	Main findings
Treatment evaluation	Wang et al. [31]	PE	MCS 7 VS/UWS 7	SCS	PE increased as compared to the baseline during SCS turning on
	Hermann et al. [23]	PE, KCC, Spectral entropy	EMCS 4 MCS 32 VS/UWS 24	tDCS	PE in the theta-alpha band showed a trend towards an increase in responders as compared to non-responders in the same parietal region

*HC* healthy control, *DOC* disorders of consciousness, *MCS*   minimally conscious state, *VS/UWS* vegetative state/unresponsive wakefulness state, *CS* conscious patients, *BIS* bispectral index, *ApEn* approximate entropy, *LZC* Lempel–Ziv Complexity, *PE* permutation entropy, *KCC* Kolmogorov-Chaitin complexity, *SCS* spinal cord stimulation, *tDCS*  transcranial direct current stimulation

**Table 3:** EEG networks in disorders of consciousness

Clinical application	Literature	Measurements	Characteristics	Subjects (numbers)	Main findings
Diagnosis	Davey et al. [39]	Functional connectivity	Coherence	VS/UWS 1	Coherence in the right (more heavily damaged) hemisphere was lower overall than in the left hemisphere in the frontal and parieto-occipital regions
	Pollonini et al. [41]	Functional connectivity	Coherence, Granger causality	MCS 7 SND 9	SND consistently formed a larger number of connections compared to MCS in all frequency bands SND formed significantly more connections from bilateral temporal, central and parieto-occipital regions into frontal areas at beta band, and from left temporal into parieto-occipital regions at delta band
	Wu et al. [34]	Functional connectivity	Cross-approximate entropy	HC 30 MCS 20 VS/UWS 30	Interconnection of local and distant cortical networks in MCS was superior to VS/UWS
	Leon-Carrion et al. [38]	Functional connectivity	Coherence, Granger causality	MCS 7 SND 9	MCS showed frontal cortex disconnection from other cortical regions, whereas SND showed a higher number of functional connections between the frontal and parietal/occipital regions
	Lehembre et al. [20]	Functional connectivity	Imaginary coherence, phase lag index	MCS 18 VS/UWS 10	Higher frontal-to-posterior connectivity in MCS as compared to the VS/UWS in the theta band
	Fingelkurts et al. [53]	Oscillatory microstates	Oscillatory microstate types, probability of occurrence and duration	MCS 7 VS/UWS 14	Occurrence and duration of delta-, theta- and slow-alpha-rhythmic microstates were associated with unawareness, whereas occurrence and duration of fast alpha-rhythmic microstates were associated with consciousness
	King et al. [42]	Functional connectivity	wSMI	HC 14 CS 24 MCS 68 VS/UWS 75	VS/UWS presented significantly lower wSMI than MCS, CS and HC wSMI was less impaired over frontal than posterior regions in VS/UWS wSMI increased with consciousness, primarily over centro-posterior regions
	Sitt et al. [13]	Functional connectivity	wSMI	HC 14 CS 24 MCS 68 VS/UWS 75	Comparing to average spectrum, complexity and global responses, wSMI was the most discriminative measure for separating VS/UWS from MCS and CS
	Chennu et al. [16]	Functional connectivity, Graph theory analysis	dwPLI, clustering coefficient, characteristic path length, modularity, participation coefficient and modular span	HC 26 MCS 19 VS/UWS 13	Significantly higher levels of clustering and shorter path lengths in alpha band in HC compared to patients Standard deviations of participation coefficients of networks nodes were significantly higher at delta and theta and lower at alpha in patients Modular span of the largest module in alpha networks in HC was higher than in patients
	Cavinato et al. [35]	Functional connectivity	Coherence	HC 15 MCS 14 VS/UWS 12	HC and MCS showed higher short-range parietal and long-range fronto-parietal coherences in gamma frequencies, while VS/UWS had a lower connected network in alpha frequency in the posterior regions, and higher and widespread theta coherence
	Thul et al. [40]	Functional connectivity	STEn	HC 24 MCS 7 VS/UWS 8	Fronto-posterior feedback interaction was decreased in accordance to the severity of the DOC (VS/UWS > MCS > HC) and allowed for a reliable differentiation of MCS and VS/UWS from HC
	Chennu et al. [52]	Functional connectivity, Graph theory analysis	dwPLI, clustering coefficient, characteristic path length modularity, participation coefficient and modular span	HC 26 LIS 4 EMCS 11 MCS + 49 MCS- 17 VS/UWS 23	Presence of hub nodes in the alpha network identified presence of consciousness in patients misdiagnosed as VS/UWS based on clinical consensus
	Stefan et al. [19]	Microstate and Graph theory analysis	Probability of occurrence, duration, coherence, wSMI, symbolic transfer entropy, clustering coefficient and path length	MCS 11 VS/UWS 51	Microstate D in the alpha frequency band performed best at distinguishing MCS from VS/UWS Coherence in the alpha and beta, wSMI in delta, theta and alpha, transfer entropy in all frequency bands distinguished VS/UWS and MCS Characteristic path length and clustering coefficient successfully classified patients into VS/UWS
	Naro et al. [15]	Functional connectivity	dwPLI	MCS 15 VS/UWS 17	dwPLI was significantly smaller in VS/UWS than MCS at alpha and gamma bands
	Bai et al. [43]	Functional connectivity	Quadratic phase self-coupling	MCS 20 VS/UWS 31	Quadratic phase self-coupling at the delta, theta and alpha bands were closely correlated with patients' CRS-R scores
	Cacciola et al. [51]	Functional connectivity, Graph theory analysis	Peak lagged phase synchronization, small-worldness, modularity, and structural consistency, characteristic path length, average clustering coefficient, global efficiency, local efficiency, node betweenness centrality, and local-community-paradigm correlation	MCS 13 VS/UWS 12	Decreased interhemispheric fronto-parietal connectivity in VS/UWS compared to MCS Increased values of local-community-paradigm correlation, higher clustering coefficient and local efficiency in VS/UWS compared to MCS
	Rizkallah et al. [49]	Functional connectivity, Graph theory analysis	Phase locking value, clustering coefficient and participation coefficient	HC 21 6 EMCS 29MCS + 17 MCS − 9 VS/UWS	Consciousness level decreased with increasing clustering coefficient and decreasing participation coefficient values Large-scale functional brain networks showed decreasing integration with lower level of consciousness
	Lee et al. [118]	Functional connectivity	Phase lag index pair correlation function phase lag entropy	HC 73 drug-induced unconsciousness 45 MCS 15 VS/UWS 27	Mean phase lag entropy distinguished conscious (HC and MCS) and unconscious states (anesthetized and VS/UWS) Mean phase lag entropy in MCS was significantly higher than in VS/UWS Overall communication complexity of the MCS brain was at a similar level compared to HC, but the communication structure was inefficient and far from the critical state, thus reducing sensitivity to external stimuli
	Cai et al. [50]	Functional connectivity Graph theory analysis	Phase lag index, multiplex clustering coefficient, participation coefficient	HC 23 MCS 19 VS/UWS 35	DOC showed a decreasing trend of segregation The multiplex network measures (positively or negatively) correlated with the patients’ CRS-R scores Disappearance of hub regions in DOC multiplex networks, particularly in the frontal and parietal-occipital areas

Clinical application	Literature	Measurements	Characteristics	Subjects (numbers)	Follow-up times	Main findings
Prognosis	Fingelkurts et al. [44]	Functional connectivity	Microstructural connectivity	VS/UWS 14	6 months post brain injury	The number and strength of cortical functional connections between EEG segments were higher in patients who recovered consciousness compared with those who did not recover
	Schorr et al. [45]	Functional connectivity	Coherence	HC 24 MCS 15 VS/UWS 58	12ths	High parietal delta and theta, and high fronto-parietal theta and alpha coherence appeared to provide strong early evidence for recovery from VS/UWS
	Chennu et al. [52]	Functional connectivity, Graph theory analysis	dwPLI, clustering coefficient, characteristic path length modularity, participation coefficient and modular span	HC 26 LIS 4 EMCS 11 MCS + 49 MCS − 17 VS/UWS 23	12 months	Modularity and clustering coefficients of delta networks were higher in patients with positive outcomes Network metrics correlated well with brain metabolism in PET
	Stefan et al. [19]	Functional connectivity, Graph theory analysis	Probability of occurrence, duration, coherence, wSMI, STEn, clustering coefficient and path length	MCS 11 VS/UWS 51	589.26 ± 1125.32 days	Imaginary part of coherence in the beta band was greater for patients who improved STEn both in the delta and alpha bands was successful at predicting outcome wSMI in th mon e alpha band exhibited the most notable prognostic power Clustering coefficients, calculated from beta and alpha bands performed best at classifying patients into the two outcome categories: VS/UWS or dead, MCS or EMCS
	Bai et al. [43]	Functional connectivity	Quadratic phase self-coupling	MCS 20 VS/UWS 31	3 months	Lower frontal quadratic phase self-coupling at the theta band indicated higher probability of consciousness recovery

Clinical application	Literature	Measurements	Characteristics	Subjects (numbers)	Treatment	Main findings
Treatment evaluation	Williams et al. [48]	Functional connectivity	Coherence	three diverse patients with known zolpidem response	Zolpidem	6–10 Hz spectral peak with high spatial coherence across intra- and inter-hemispheric brain regions is a predictor of zolpidem responsiveness
	Naro et al. [24]	Functional connectivity	Coherence	HC 15 MCS 12 VS/UWS 14	tACS	tACS entrained theta, gamma oscillations and strengthened connectivity within frontoparietal networks in all HC and, partially, all MCS, and some VS/UWS
	Naro et al. [25]	Functional connectivity	Coherence	HC 10 MCS 10 VS/UWS 10	otDCS	Coherence of fronto-parietal networks at theta and gamma bands increased in MCS but not VS/UWS after otDCS cerebellar-cerebral connectivity modulation
	Bai et al. [26]	Functional connectivity	Coherence, bicoherence	MCS 11	SCS	Bicoherence showed decreased coupling within delta after 70 Hz SCS, and reduction of coupling between delta and gamma with 5 Hz and 100 Hz SCS
	Bai et al. [46]	Functional connectivity	Coherence	MCS 8 VS/UWS 9	tDCS	After real tDCS, fronto-parietal coherence significantly increased in the theta band and decreased in the gamma band in MCS
	Cavinato et al. [47]	Functional connectivity	Coherence	MCS 12 VS/UWS 12	tDCS	Increase of coherence of the frontal and parietal regions at alpha and beta after tDCS in MCS
	Hermann et al. [23]	Functional connectivity	wSMI	EMCS 4 MCS 32 VS/UWS 24	tDCS	Responses to tDCS were characterized by an increase of functional connectivity in the theta-alpha band (4–10 Hz) A parieto-occipital cluster with increased values in responders as compared to non-responders

*HC* healthy control, *DOC* disorders of consciousness, *EMCS* emerged form minimally conscious state, *MCS* minimally conscious state, *MCS +* minimally conscious state plus, *MCS−* minimally conscious state minus, *VS/UWS* vegetative state/unresponsive wakefulness state, *LIS* lock-in syndrome, *CRS-R* JFK Coma Recovery Scale-Revised, *SND* severe neurocognitive disorders, *CS* conscious patients, *PET* fluoro-2-deoxy-d-glucose–positron emission tomography, *otDCS* oscillatory transcranial direct current stimulation, *SCS* spinal cord stimulation, *tACS * transcranial alternating current stimulation, *tDCS* transcranial direct current stimulation, *wSMI* weighted symbolic mutual information, *dwPLI*  debiased weighted phase lag index, *STEn* symbolic transfer entropy

**Table 4:** EEG sleep patterns in disorders of consciousness

Clinical application	Literature	Subjects (numbers)	Main findings
Diagnosis	Oksenberg et al. [58]	VS/UWS 10	VS/UWS patients had a significant reduction in the phasic activities of rapid eye movement sleep The amount of sleep activities was unrelated to recovery from the clinical condition
	Isono et al. [59]	VS/UWS 12	In 8 VS/UWS patients, a clear sleep wake cycle was observed, and five of them showed diffuse low voltage slow waves, with ripple-like activities shortly after the start of sleep
	Landsness et al. [57]	MCS 6 VS/UWS 5	Observed behavioral, but no electrophysiological, sleep wake patterns in VS/UWS, while there were near-to-normal patterns of sleep in in MCS
	Cologan et al. [61]	MCS 10 VS/UWS 10	VS/UWS preserved sleep spindles, slow-wave sleep, and rapid eye movement sleep
	Malinowska et al. [64]	LIS 1 MCS 20 VS/UWS 11	MCS and LIS had significantly more sleep spindles, delta waves and cycles of light and deep sleep than VS/UWS Traumatic patients were more likely to show preserved EEG sleep-like activities
	Forgacs et al. [60]	EMCS 13 MCS 23 VS/UWS 8	Patients presenting vertex waves and sleep spindles had significantly higher best CRS-R scores than the others
	Mouthon et al. [56]	CS 5 EMCS 1 MCS 4	Children with DOC showed a global reduction in sleep-wave activities regulation over parietal brain areas
	Wislowska et al. [55]	HC 26 MCS 17 VS/UWS 18	Prevalence of sleep spindles and slow waves did not systematically vary between day and night in patients Day-night changes in EEG power spectra and signal complexity were revealed in MCS but not VS/UWS The more parietal spindles during night-time, the higher CRS-R scores
	Pavlov et al. [62]	VS/UWS 15	Sleep stage N1 was observed in 13 patients, N2 in 14 patients, N3 in 9 patients and REM sleep in 10 patients. Sleep spindles were found in five patients Stage N2 was mostly characterized by typical K-complexes, but spindles were observed only in five patients, and their density was low
	Rossi Sebastiano et al. [63]	MCS 36 VS/UWS 49	The presence of slow-wave sleep was the most appropriate factor for classifying patients as VS/UWS or MCS Duration of slow-wave sleep was demonstrated as the main factor that significantly correlated with patients' CRS-R scores
	Wielek et al. [66]	MCS 11 VS/UWS 12	MCS were more likely to wake up during the day and had more complex patterns of sleep–wake stages at night, while VS/UWS did not show any accumulation of specific conditions during the day or night
	Zieleniewska et al. [65]	EMCS 5 MCS 6 VS/UWS 8	Power and detrended fluctuation analysis of sleep spindles, detrended fluctuation analysis of slow waves and times of deep sleep were higher in the MCS/EMCS groups than VS/UWS

Clinical application	Literature	Subjects	Follow-up times	Main findings
Prognosis	Cologan et al. [61]	MCS 10 VS/UWS 10	6 months	Occurrence of sleep spindles related to clinical improvement within 6 months

*HC* healthy control, *DOC* disorders of consciousness, *EMCS* emerged form minimally conscious state, *MCS* minimally conscious state, *VS/UWS* vegetative state/unresponsive wakefulness state, *LIS* lock-in syndrome, *CRS-R* FK Coma Recovery Scale-Revised, *CS* conscious patients

**Table 5:** Evoked EEG potential measurements in disorders of consciousness

Clinical application	Stimulus pathway	Literature	Stimulus	Components	Subjects (numbers)	Main findings
Diagnosis	Auditory	Kotchoubey et al. [67]	Oddball 1 Standard: sine tones (1200 Hz) Deviant: sine tones (700 Hz) Oddball 2 Standard: harmonic chord music with probability of target stimulus 0.2 Deviant: harmonic chord music with probability of rare stimulus 0.8 Oddball 3 Two natural sounds were presented: standard: /o/ deviant: /i/	MMN N100 P200 P300	CS 22 MCS 38 VS/UWS 38	Low-level cortical processing presented in all MCS, but also in all VS/UWS with at least minimally preserved thalamo-cortical connections
		Perrin et al. [69]	Standard: OFN Deviant: SON	P300	HC 5 LIS 4 MCS 6 VS/UWS 5	P300 was observed in response to SON in all LIS, in all MCS, and in 3/5 VS/UWS P300 latency was significantly delayed for MCS and VS/UWS compared with HC
		Schnakers et al. [72]	8 first SON and seven other unfamiliar first names presented in a randomized order Passive: just listen Active: count SON or unfamiliar first names	P300	HC 12 MCS 14 VS/UWS 8	Like HC, MCS presented a larger P300 to SON, in both passive and active conditions。 P300 to target stimuli was higher in the active than in the passive condition No difference of P300 between SON and unfamiliar names in VS/UWS
		Fischer et al. [68]	Oddball stimulation paradigm including standard tones, duration-deviant tones and novel stimuli Standards and deviants were tone-bursts of 800 Hz, lasting 75 ms for the standards and 30 ms for the deviants, novel stimuli were SON	MMN N100 P300	MCS 11 VS/UWS 16	MMN was present in only five patients (two VS/UWS, three MCS) N100 was present in 12 patients (4 VS/UWS, 8 MCS) Novelty P300 was present in seven patients (three VS/UWS, four MCS)
		Risetti et al. [73]	Standard: tone bursts of 800 Hz; Deviant: tone bursts of 1 kHz; Novel stimulus: SON Passive: just listen Active: count the own name	P300	MCS 3 VS/UWS 8	Larger P300 and a wider spatial distribution in active vs. passive listening only in MCS but not VS/UWS
		Sitt et al. [13]	Low-pitched 350, 700 and 1400 Hz tones, hereafter sound X; High pitched 500, 1000 and 2000 Hz tones, hereafter sound Y; Standard: 80% XY/20% XX Deviant: 80% XX/20% XY	P300 MMN	HC 14 CS 24 MCS 68 VS/UWS 75	MMN discriminated VS/UWS from CS and MCS from CS, but did not discriminate VS/UWS from MCS P300 topography failed to discriminate VS/UWS from MCS
		Li et al. [70]	Oddball 1 Standard: 1000 Hz tone Deviant: SON Oddball 2 Standard: subject’s derived name Deviant: SON	P300	HC 17 MCS 5 VS/UWS 6 Coma 2	All MCS had a P300 in the first paradigm and the majority of them in the second paradigm. Most VS/UWS had no P300
		Balconi and Arangio [78]	Incongruous words sequence: [cherry, apple, melon, cuttle]; or congruous words sequence: [cherry, apple, melon, grapes]	N400	MCS 11 VS/UWS 7	VS/UWS showed a delayed N400 in comparison with MCS in the incongruous condition N400 peak increased in the fronto-central cortical areas in the incongruous condition in all patients
		Rohaut et al. [81]	Standard: identical four similar sounds (1000 or 2000 Hz Deviant: a different fifth sound	N400 LPC	HC 20 MCS 15 VS/UWS 15	N400-like ERP components could be observed in VS/UWS, MCS and HC, only MCS and HC showed a LPC response
		Real et al. [119]	Standard: high (440 + 880 + 1760 Hz) tones; Deviant: low (247 + 494 + 988 Hz) tones; Passive: just listen Active: count the odd tones	P300	HC 14 MCS 16 VS/UWS 29	P300 was higher in HC than in patients, but did not discriminate between VS/UWS and MCS
		Beukema et al. [77]	Standard: 400 signal-correlated noise stimuli; Deviant: 400 words (100 related word-pairs,100 unrelated word-pairs)	N400	HC 17 MCS 8 VS/UWS 8	No difference in auditory processing between VS/UWS and MCS 4 MCS and 3 VS/UWS exhibited significant speech vs. noise effects, without difference between MCS and VS/UWS
		Sergent et al. [82]	An auditory cue (either SON, OFN or a non-vocal control) was played on the left or right side, followed by a target (sinusoidal tones: 500, 1000 or 2000 Hz) on same or other side Patients were instructed to imagine squeezing their hand on the target side	P300 Contingent negative variation Lateralized readiness potential	HC 15 CS 1 MCS 8 VS/UWS 4	P300 effect in most but not all HC (9/15), in 4/8 MCS and 1/4 VS/UWS Significant contingent negative variation was observed for all HC, for the CS, for 5/8 MCS and 3/4 VS/UWS Significant lateralized readiness potential existed in 8/15 HC, in 2/8 MCS patients, but none of the VS/UWS
		Erlbeck et al. [76]	MMN paradigm: three-component harmonic sounds of 440 + 880 + 1760 Hz, standard and deviant stimuli with different duration N400 words paradigm: semantically related and unrelated work-pairs N400 sentence paradigm: short sentences ended with a correct or incorrect word,	MMN N400 LPC	EMCS 3 MCS 3 VS/UWS 13	Majority patients (n = 15) show no response to stimulation MMN was identified in 2 patients, and LPC was identified in 2 patients
		Rivera-Lillo et al. [71]	SON and 7 other unfamiliar first names	P300	HC 10 MCS 3 VS/UWS 10	MCS and VS/UWS showed reduced modulation of spectral activity in the delta band, which indicated dissociation in the P300 related neural networks
		Kempny et al. [120]	SON, other names and time-reversed other names, two SON trials were randomly inserted in the two auditory blocks (other and reversed names)	P300 negative ~ 700 ms	HC 12 MCS 11 VS/UWS 5	3 MCS, and 1 VS/UWS had significant difference in EEG response to SON versus other names with ERP latencies: ~ 300 ms and ~ 700 ms
		Wu et al. [121]	Standard: meaningless neutral sound (the interjection “ah”); Deviant: same sound with positive or negative affective prosody; four different voices from the validated battery of vocal emotional expressions	N100 P300 LPC	MCS 22 VS/UWS 20	MCS showed significant N100 and P300, but VS/UWS only showed N100 No LPC was detected in these patients
	Visual	Wijnen et al. [83]	Repeatedly visual stimuli (visual localization, comprehension of written commands, and object manipulation) and flash VEP	VEP	HC 22 VS/UWS 11	VEP amplitudes were smaller, and latencies were longer in VS/UWS compared to HC
		Naro et al. [84]	Associative stimulation combining transcranial magnetic stimulation with visual stimulation through transorbital alternating current stimulation	VMI and P300	MCS 7 VS/UWS 7	MCS showed VMI and P300, whereas VS/UWS showed no VMI
	BCI	Cruse et al. [99]	Motor imagination of right-hand and toe movements on command	EEG respond traces	HC 12 VS/UWS 16	3/16 VS/UWS could repeatedly and reliably generate appropriate EEG responses to two distinct motor imagination commands
		Lule et al. [87]	Four choice command following paradigm	P300	HC 16 LIS 2 MCS 13 VS/UWS 3	EEG-based BCI detected command following in DOC patients and functional communication in LIS Only 1/13 MCS 0/3 VS/UWS showed command following
		Pan et al. [90]	Own photo and unfamiliar photo flashed in a random order	Visual hybrid BCI combining P300 and steady-state evoked potential	HC 4 LIS 1 MCS 3 VS/UWS 4	1/4 VS, 1/3 MCS and 1 LIS were able to selectively attend to their own or the unfamiliar photos
		Li et al. [122]	Three tasks: number recognition, number comparison, and mental calculation	P300 and steady-state evoked potential	EMCS 2 MCS 3 VS/UWS 6	2/6 VS/UWS, 1/3 MCS and 2 EMCS had significant P300 and VEP
		Coyle et al. [98]	Imagined hand movement versus toe wiggling	EEG response traces	MCS 4	MCS have the capacity to operate a simple BCI-based communication system, even without any detectable volitional control of movement
		Wang et al. [123]	Audiovisual stimuli: the color of the flashing button and the corresponding spoken word were broadcasted	P300	LIS (MCS) 1	A LIS patient was found misdiagnosed as MCS
		Xiao et al. [86]	Standard: background noise Deviant: a clap	P300 MMN Auditory startle	HC 5 EMCS 1 MCS 6 VS/UWS 14	Three VS/UWS patients did not respond to CRS-R but to BCI
		Wang et al. [92]	Audiovisual stimuli: six situation-orientation questions like “Am I touching my ear/nose right now?”	EEG respond traces	MCS 5 VS/UWS 8	4 VS/UWS and three MCS were unresponsive in the CRS-R assessment but responsive in the BCI-based assessment, and four of those improved later in the CRS-R-based assessment
		Xiao et al. [88]	Ball flashing randomly	EEG respond traces	HC 5 LIS 1 EMCS 1 MCS 5 VS/UWS 8	2 MCS and one LIS showed visual fixation in both CRS-R and BCI, one VS/UWS did not show behavior in CRS-R but in BCI
		Xiao et al. [89]	Visual pursuit following the moving picture	P300 visual pursuit	HC 5 LIS 1 EMCS 1 MCS 6 VS/UWS 6	7 patients (4 VS/UWS, three MCS) who did not exhibit visual pursuit in CRS-R were responsive to the moving target in BCI
		Pan et al. [91]	Focus on the crying or laughing movie clip and count the flashes	P300	HC 8 MCS 5 VS/UWS 3	2 MCS, one VS/UWS and all HC had abilities of emotion recognition and command following
		Xie et al. [93]	Gaze-independent audiovisual BCI system: semantically congruent and incongruent audiovisual number stimuli	P300 N400 LPC	HC 10 MCS 3 VS/UWS 5	2 MCS and one VS followed commands and recognized numbers, like HC
		Annen et al. [96]	Mentally count the stimuli presented on the right wrist	P300	MCS 4 VS/UWS 8	1 MCS patient showed 'covert command following' during the active tactile paradigm and showed a higher cerebral glucose metabolism within the language network when compared with the other patients without 'covert command-following' but having a cerebral glucose metabolism indicative of MCS
		Curley et al. [100]	Four different motor imagery tasks included ‘tennis’ (swinging a tennis racket with one hand), ‘open/close right (left) hand’, ‘navigate’ (walking through one’s house), and ‘swim’	Spectrum of EEG responses	HC 15 EMCS 9 MCS 21 VS/UWS 4	9/21 patients (1 VS/UWS, 5 MCS, 3 EMCS) with EEG evidence of command-following also demonstrated functional MRI evidence of command-following 9 patients (2 VS/UWS, 6 MCS, 1 EMCS) with EEG command-following capacity showed no behavioral evidence of a communication by CRS-R 5/9 patients (two VS/UWS, one MCS, two EMCS) with statistically indeterminate responses to one task showed a positive response after accounting for variations in overall background state
		Spataro et al. [97]	Count vibrotactile stimuli delivered to the left or right wrist	EEG respond traces	HC 6 VS/UWS 13	4 VS/UWS patients demonstrated clear EEG-based indices of task following in one or both paradigms, which did not correlate with clinical factors
		Guger et al. [95]	VT2: two vibro-tactile stimulators fixed on the patient's left and right wrists VT3: three vibro-tactile stimulators fixed on both wrists and on the back Mentally count either the stimuli on the left or right wrist	P300	VS/UWS 12	Grand average VT2 accuracy across all patients was 38.3%, and the VT3 accuracy was 26.3% Two patients achieved VT3 accuracy ≥ 80% and went through communication testing

Clinical application	Stimulus pathway	Literature	Stimulus	Components	Subjects (numbers)	Follow-up times	Main findings
Prognosis	Auditory	Kotchoubey et al. [67]	Oddball 1 Standard: sine tones (1200 Hz) Deviant: sine tones (700 Hz) Oddball 2 Standard: harmonic chord music with probability of target stimulus 0.2 Deviant: harmonic chord music with probability of rare stimulus 0.8 Oddball 3 Two natural sounds were presented: standard: /o/ deviant: /i/	MMN N100 P200 P300	CS 22 MCS 38 VS/UWS 38	6 months	Presence of MMN significantly correlated with the 6-month outcome
		Cavinato et al. [75]	Oddball 1 Standard: sine tone of 1000 Hz Deviant: sine tone of 2000 Hz Oddball 2 Standard: tone bursts of 1000 Hz Deviant: SON Oddball 3 Standard: tone bursts of 1000 Hz Deviant: Other First Name	P300	HC 10 MCS 6 VS/UWS 11	no information available	P300 latency predicted the recovery of consciousness from VS/UWS to MCS
		Faugeras et al. [124]	Series of five complex 50-ms-duration sounds with an intensity of 70 dB and 150 ms between sounds; each sound was composed of three sinusoidal tones (either 350, 700, and 1400 Hz; or 500 Hz, 1000 Hz, and 2000 Hz)	Short-interval violations Long-term violations	HC 10 VS/UWS 22	6 months	Two VS/UWS with a positive ERP test showed unequivocal clinical signs of consciousness within the 3–4 days following ERP recording In the 20 remaining patients with a negative result, early recovery of consciousness was observed in only 2 cases within the first week
		Steppacher et al. [79]	Oddball 1 Standard: 1000 Hz sine tones Deviant: 1500 Hz sine tones Oddball 2 Standard: correct sentence Deviant: senseless sentence	N400 P300	MCS 39 VS/UWS 53	2–14 years after discharge	Presence of N400 (rather than P300) predicted recovery and restoration of communication ability
		Castro et al. [74]	Music condition: music, SON and other’s first name in random order; Control condition: music-like noise stimulus	N200 P300	MCS 6 VS/UWS 7	6 months	All patients who developed a significant N200 and/or P300 event-related potential in the music condition showed a favorable outcome
		Formisano et al. [125]	Sentences task: last word in 100 sentences was congruent while that in the other 100 sentences was semantically incongruent	N400	MCS 8 VS/UWS 7	1 year	N400 was detectable and significant in those recovered from MCS with no aphasia during follow-up Presence/absence of the N400 was consistent with the brain lesion side
		Steppacher et al. [80]	Oddball 1 Standard: 1000 Hz sine tone Deviant: 1500 Hz sine tone Oddball 2 Standard: correct sentence Deviant: senseless sentence	N400 P300 N100 MMN	MCS 43 VS/UWS 59	2-15 years	The highest predicted chance of recovery with 97% was reached for MCS with both detectable N400 and P300 The lowest predicted recovery chance (around 10%) unfolds for VS/UWS with neither a N400 nor a P300
	Visual	Wijnen et al. [83]	Visual localization, comprehension of written commands, and object manipulation	VEP	HC 22 VS 11	2–3 years (long term outcome)	Initial VEP latencies were of significant prognostic value in predicting long-term outcome
	BCI	Spataro et al. [97]	Count vibrotactile stimuli delivered to the left or right wrist	EEG respond traces	HC 6 VS/UWS 13	6 months	The efficacy of somatosensory discrimination strongly correlated with the clinical outcome at 6 months
		Pan et al. [94]	Visual photograph paradigm, number paradigm, or audiovisual paradigm	P300, steady-state visual evoked potential	MCS 33 VS/UWS 45	3 months	15/18 VS/UWS with command following regained consciousness 5 of the other 27 VS/UWS without command following regained consciousness 14/16 MCS with command following showed improvements in their CRS-R scores 4 of the other 17 MCS without command following had improved CRS-R

*HC* healthy control, *DOC* disorders of consciousness, *LIS*  lock in syndrome, *EMCS* emerged form minimally conscious state, *MCS* minimally conscious state, *VS/UWS* vegetative state/unresponsive wakefulness state, *LIS* lock-in syndrome, *CRS-R* JFK Coma Recovery Scale-Revised, *CS* conscious patients, *SON* subject’s own name, *OFN* other first name, *BCI* brain computer interface, *VEP* visual evoked potentials, *MMN* mismatch negativity, *BAEPs* brainstem auditory evoked potentials, *MLAEPs*  middle-latency evoked potentials, *LPC*  late positive components, *VMI* visuomotor integration

**Table 6:** TMS-EEG measurements in disorders of consciousness

Clinical application	Literature	Characteristics	Subjects (numbers)	Main findings
Diagnosis	Rosanova et al. [104]	TEP GMFP	LIS 2 MCS 5 VS/UWS 5	In VS/UWS, TMS triggered a simple, local response In MCS, TMS invariably triggered complex responses that sequentially involved distant cortical areas ipsi- and contralateral to the site of stimulation, similar to responses in LIS
	Casali et al. [110]	PCI	LIS 2 EMCS 6 MCS 6 VS/UWS 6	PCI range: VS/UWS 0.19–0.31 LIS 0.51–0.62 MCS 0.32–0.49 EMCS 0.37–0.52 Wake 0.44–0.67
	Ragazzoni et al. [107]	TEP	HC 5 MCS 5 VS/UWS 8	TEP results suggest that cortical reactivity and connectivity are severely impaired in all VS/UWS, whereas in most MCS, TEP are preserved but with abnormal features
	Gosseries et al. [126]	TEP	HC 8 VS/UWS 3	TEP are genuine cortical responses detectable only when preserved cortical tissue is stimulated
	Formaggio et al. [127]	TEP with time–frequency analysis	HC 5 MCS 1 VS/UWS 4	Early synchronization, particularly over motor areas for alpha and beta and over the frontal and parietal electrodes for beta power in DOC patients No relevant modification in slow rhythms (delta and theta) after TMS in DOC patients
	Casarotto et al. [112]	PCI PCImax	LIS 5 EMCS 9 MCS + 17 MCS − 21 VS/UWS 43	PCImax was lower in MCS (range = 0.27–0.55) as compared to conscious brain-injured patients PCImax had a sensitivity of 94.7% in detecting minimal signs of consciousness VS/UWS had 3 different subgroups: a “no response” subgroup (PCImax = 0) of 13 patients (30%), a “low-complexity” subgroup (PCImax < 0.31) of 21 patients (49%), and a “high-complexity” subgroup (PCImax > 0.31) of 9 patients (21%)
	Bodart et al. [113]	PCI PCImax	LIS 2 EMCS 2 MCS 11 VS/UWS 9	PCI was highly consistent with FDG–PET in classifying MCS vs. VS/UWS Four patients (VS/UWS, diagnosed by CRS-R) had high PCI and preserved FDG-PET
	Rosanova et al. [108]	TEP PCI	HC 20 VS/UWS 16	VS/UWS had cortical OFF-periods, similar to those in HC during sleep

Clinical application	Literature	Characteristics	Subjects (numbers)	Main findings
Prognosis	Casarotto et al. [112]	PCI PCImax	LIS 5 EMCS 9 MCS + 17 MCS − 21 VS/UWS 43	Concerning the outcome at 6 months, 6 of 9 (1 unknown) high-complexity (PCImax > 0.31) VS/UWS transitioned to a behavioral MCS, whereas such transition was observed in 5 of 21 (2 unknown) low-complexity VS/UWS (PCImax < 0.31)
	Bodart et al. [113]	PCI PCImax	LIS 2 EMCS 2 MCS 11 VS/UWS 9	1 VS/UWS with relatively preserved whole right hemisphere metabolism but PCI < 0.31 did not show improvement and remained VS/UWS after a follow-up of 5 years

Clinical application	Literature	Characteristics	Treatment	Subjects (numbers)	Main findings
Treatment evaluation	Bai et al. [109]	GMFP	tDCS	MCS 7 VS/UWS 9	tDCS induced global TEP responses in MCS but only local responses in VS/UWS
	Bai et al. [114]	TEP GMFP PCI	rTMS	HC 5 MCS 1	Along with consciousness recovery in one MCS, TEP and PCI tended to become gradually similar to those of HC

*HC* healthy control, *DOC* disorders of consciousness, *EMCS* emerged form minimally conscious state, *MCS* minimally conscious state, *VS/UWS* vegetative state/unresponsive wakefulness state, *LIS* lock-in syndrome, *CRS-R* JFK Coma Recovery Scale-Revised, *TEP* TMS evoked Potential, *GMFP* global mean field power, *PCI* perturbational complexity index, *PCImax* max PCI, *tDCS* transcranial direct current stimulation, *TMS* transcranial magnetic stimulation, *rTMS* repetitive transcranial magnetic stimulation

## Spontaneous electroencephalogram

### Resting state

#### Spectral power

Spontaneous EEG oscillations can be divided into several frequency bands, which are generally as follows: delta (1.5–3.5 Hz), theta (3.5–7.5 Hz), alpha (7.5–12.5 Hz), beta (12.5–30 Hz) and gamma (> 30 Hz), according to their specific roles in different brain functions [9, 10]. The power of these frequency bands is typically highly abnormal in DOC patients (Table 1). Enhanced delta and suppressed alpha activities have been the highlighted markers of low consciousness level [11]. The delta power in DOC patients is higher than in healthy controls (HC) [12, 13], and in MCS patients [14]. Spectral power is dominated by the delta band in nearly 80% of VS/UWS patients [15, 16]. In addition, power in the theta band is increased in VS/UWS patients compared with HC [12] and in MCS patients compared with HC or conscious patients [17]. However, MCS patients show higher theta power compared with VS/UWS patients [14].

The depression of alpha power in VS/UWS patients is much stronger than in MCS patients [18]. The alpha power in MCS patients is significantly higher than in VS/UWS patients [19, 20], sometimes even twice as high [15]. Alpha power is higher in the central, parietal and occipital regions in MCS patients compared with VS/UWS patients [11, 18]. Residual consciousness of DOC patients tightly correlates with alpha power in the frontal and parietal networks [16, 21]. Furthermore, increase of alpha power over time is associated with the chance of consciousness recovery [19, 22, 23].

High-frequency bands (beta and gamma) have been rarely considered in DOC studies. One study found significantly higher low beta (12–17.75 Hz) power in MCS compared with VS/UWS [14], but in the whole beta band, normalized beta power does not discriminate between MCS and VS/UWS [13]. Gamma power in DOC patients is lower than in HC [18]. Gamma activity has a critical role in assessing DOC patients’ responses to specific brain stimulation: transcranial alternating current stimulation [24], transcranial direct current stimulation [25] and spinal cord stimulation [26] can all significantly increase gamma power.

Quantified indexes based on spectral power in more than one frequency band are often used for diagnosis, treatment monitoring and outcome prediction of DOC patients. Ratios between lower frequencies (delta and theta) and higher frequencies (alpha and beta) show close association with regional glucose metabolism in MCS but not VS/UWS [27]. Moreover, there is a positive correlation between the power ratio (above 8 to below 8 Hz) and the CRS-R score, and the peak frequency of the spectrum is also strongly related to the CRS-R score [12].

#### Non-linear dynamics

##### Non-linearity in the time domain

In time domain analyses, VS/UWS patients always show the lowest EEG complexity, while MCS patients have higher but still moderately lower complexity than HC [28]. Approximate entropy (ApEn) is one of the frequently used non-linear indices for capturing consciousness-related EEG complexity. The ApEn values of VS/UWS patients are commonly lower than in HC (sensitivity: 95%, specificity: 100%) [29]. ApEn values in VS/UWS are also lower than in MCS over a broad frequency band [19], which is consistent with the Lempel–Ziv complexity analysis [28]. In addition, ApEn and Lempel–Ziv complexity are closely associated with outcome [28]. Most patients with the lowest ApEn values die or remain in persistent VS/UWS at 6-month follow-up, while patients with higher ApEn values are either in MCS, or partially or completely recovered [29].

Permutation entropy is effective in differentiating MCS from VS/UWS [13, 19]. A comparative study on EEG markers, including three non-linear indices, showed that only permutation entropy and Kolmogorov–Chaitin complexity significantly differentiate between VS/UWS and MCS [13]. Consistently, permutation entropy is as one of the top ranking among over 20 potential biomarkers in the classification of different consciousness states of DOC patients [30]. Permutation entropy is also a sensitive index responding to brain stimulation treatment [31]. Moreover, permutation entropy in the delta and theta bands shows better performance than ApEn as a prognostic index in predicting outcome of DOC patients [19] (Table 2).

##### Non-linearity in the frequency domain

Spectral entropy is a non-linear quantitative parameter of specific spectral EEG powers. VS/UWS patients have lower spectral entropy values than MCS patients and fully conscious patients [13]. MCS exhibits periodic (over 70 min) fluctuations of spectral entropy similar to HC. In contrast, VS/UWS does not show any distinct periodic fluctuations [14]. State entropy, reaction entropy and bispectral index (BIS), which were used in detecting the depth of anesthesia, are also effective in distinguishing VS/UWS from MCS with sensitivity of 89% and specificity of 90% [32] (Table 2). The state entropy values of VS/UWS and MCS are 49% and 18% lower, respectively, than of HC. Consistently, lower consciousness levels correspond to lower BIS in DOC patients [33]. Moreover, BIS correlates positively with the CRS-R, and the 1-year outcome of DOC patients [33].

#### Brain networks

##### Functional connectivity

DOC patients with a better state of consciousness tend to show stronger functional connectivity among brain regions [34, 35] (Table 3). The prefrontal cortex plays a crucial role in the formation of consciousness [36, 37]. Disconnection between the frontal and parietal and occipital regions discriminates between MCS patients and individuals with severe neurocognitive disorders [38]. MCS has a higher phase lag index and imaginary coherence between the frontal and posterior regions than VS/UWS [20]. Coherence between the frontal and occipital regions in the ipsilesional hemisphere is lower than in the contralesional hemisphere in VS/UWS [39]. Symbolic transfer entropy analysis also shows that the feedback interaction between the frontal-parietal and frontal–temporal regions is particularly affected in DOC patients [40]. MCS patients show less connectivity between temporal and parietal-occipital regions than patients with severe neurocognitive disorders [41].

Furthermore, MCS and VS/UWS show significantly abnormal connectivity dynamics, especially in the frontotemporal, frontocentral and centro-parietooccipital regions [15]. Connectivity measured by weighted symbolic mutual information increases over central-posterior regions with increasing levels of consciousness [42].

Connectivity measured in theta, alpha and gamma bands shows significance in distinguishing consciousness levels. Compared with VS/UWS, MCS has a higher phase lag index in the alpha band [20], an increasing debiased estimator of the squared weighted phase lag index in the gamma band, and a lower coherence in the theta band [15, 35]. In addition to traditional connectivity measurements, bicoherence measures the quadratic phase coupling characteristics of the EEG oscillations within the same sources. Quadratic phase self-coupling in the delta, theta and alpha bands is closely correlated with the CRS-R score [43].

Functional connectivity measurement also showed utility in the outcome prediction of DOC patients. In a 3-month prognostic study, the number and intensity of cortical functional connections of recovery-conscious patients were higher than those of unrecovered patients [44]. The residual coherence in the parietal and frontoparietal regions provides strong evidence for early recovery of VS/UWS, with a high predictive sensitivity and specificity (parietal coherence at theta: sensitivity = 73%, specificity = 79%; frontoparietal coherence at alpha: sensitivity = 64%, specificity = 77%) [45]. Symbolic transfer entropy in the delta and alpha bands also shows a significant prognostic effect [19]. In addition, lower frontal quadratic phase self-coupling in the theta band indicates increased probability of consciousness recovery [43].

Some treatments modify the connectivity properties in DOC patients. MCS vs. VS/UWS patients show significantly different responses of frontoparietal connectivity induced by transcranial direct current stimulation or transcranial alternating current stimulation [24, 25, 46, 47]. Coherence of the prefrontal cortex can be modified by spinal cord stimulation [26]. And parieto-occipital connectivity can be modified by transcranial direct current stimulation [23]. Increasing spatial coherence within one cerebral hemisphere and between the hemispheres is also considered as a critical predictor of DOC patients responding to zolpidem [48].

##### Graph theory analysis

Graph theory analysis provides an approach to quantify the features of functional neural networks. Brain networks of DOC patients show the characteristics of impairment of global information processing (network integration) and increase of local information processing (network isolation) compared with HC [49, 50]. With a decreased consciousness level, the integration of large-scale brain function networks decreases [49], but at the same time, segmentation and local efficiency increase [51]. Path length and clustering coefficient can successfully distinguish MCS from VS/UWS [19]. DOC patients show significantly higher local efficiency, lower modularization and higher centrality in the delta and theta band networks compared with HC [16]. Furthermore, patients misdiagnosed as VS/UWS have a similarly powerful frontal lobe brain network as MCS patients: the participation coefficient can define the existence of a hub node in the alpha network, and it is possible to clarify whether a given patient was misdiagnosed as VS/UWS based on clinical consensus [52]. Finally, a support vector machine (SVM) trained by alpha participation coefficient and delta band power could diagnose VS/UWS, MCS minus, MCS plus with 74%, 100% and 71% accuracy. And a SVM trained by delta modularity and clustering coefficient was able to predict outcomes of VS/UWS and MCS with accuracy of 80% and 87%, respectively [52].

##### Microstates

Reduction of the number of EEG microstate types is associated with an altered state of consciousness (Table 3). The lack of awareness may be due to the lack of diversity of microstates in the alpha band. This highlights the importance of the fast alpha-rhythm microstate in the formation of consciousness, and also demonstrates that probability and duration of delta, theta and slow-alpha microstates are associated with unconsciousness [53]. Also, the percentage time of alpha-rhythm microstates can distinguish MCS and VS/UWS [19].

### Sleep patterns

The sequential alternation of sleep stages, the presence of sleep cycles, and characteristic sleep-stage dependent EEG patterns such as sleep spindles or slow-wave oscillations are always found in healthy subjects [54]. However, they are disturbed in DOC patients (Table 4). DOC patients do not show systematic variance of sleep spindles and slow-wave oscillations between day and night [55]. Moreover, a reduction in the build-up of slow-wave oscillations during sleep over parietal brain areas is commonly found in DOC patients, which is considered a characteristic topographical marker for network dysfunction [56].

Whether sleep EEG patterns are evident in VS/UWS patients is still unclear and a matter of ongoing debate. Some studies have suggested that most VS/UWS patients retain sleep behavior but do not have sleep EEG patterns (slow-wave/rapid eye movement (REM) sleep phases or synchronized regulation of slow-wave activity) associated with night-time sleep [57]. However, several other studies have suggested that VS/UWS patients can exhibit preserved sleep behaviors and EEG sleep patterns [58, 59]. Several sleep EEG components, such as spindles, slow-wave oscillations, and REM sleep are preserved in some VS/UWS patients [60–62]. Slow-wave sleep characteristics were demonstrated as the main factor that significantly correlates with the CRS-R score [63] and distinguishes between conscious patients (MCS and lock-in syndrome [LIS]) and VS/UWS [64, 65]. Some MCS patients have alternating non-REM (NREM)/REM sleep patterns with isotactic reduction in their night-time EEG [57], which is predictive of good recovery. A machine learning study showed that MCS patients are more likely to wake up during the day and have more complex patterns of sleep–wake stages at night, while VS/UWS patients do not show any accumulation of specific conditions during the day or night [66]. In addition, the number of sleep spindles and K-complexes in DOC patients increases with consciousness recovery over a 6-month follow-up [61].

## Evoked electroencephalogram

### Event-related potentials

#### Auditory evoked potentials

The majority of MCS and, to a lesser extent, VS/UWS have preserved cortical responses under auditory stimulation [67, 68] (Table 5). The most commonly used auditory paradigm is the oddball paradigm, which usually applies standard stimulation with a 1-kHz tone and deviation stimulation with an 800 Hz, 1200 Hz or 2 kHz tone with a period of 50–100 ms. However, due to the low levels of consciousness and cognitive ability, pure tones may not elicit any differential responses among DOC patients of different severity. When adding more cognitive content in auditory stimulation, this can provide valuable information for detecting covert cognitive ability of behaviorally unresponsive patients. In DOC studies, the oddball experiment is usually designed with a combination of the subject’s own name (SON) vs. other first name (OFN) [69]. 

The P300 in auditory stimulation paradigms is one of main components in DOC research. It could be detected in some MCS patients, but is less evident or absent in VS/UWS [70]. In the SON vs. OFN paradigm, both MCS and VS/UWS show reduced modulation of spectral activity in the delta band, which indicates dissociation in the P300-related neural networks [71]. When comparing active (count SON or OFN) and passive (just listen) task conditions, P300 of some MCS patients was higher in the active than passive conditions, suggesting their capability of voluntarily complying with task instructions like HC [72]. In contrast, no P300 differences between passive and active conditions were observed in VS/UWS. In addition, the novelty P300 in active task had a wider spatial distribution than during passive listening in MCS. There was no such effect in VS/UWS [73]. Furthermore, P300 also shows prognostic value in DOC. Patients with a present P300 in some modified paradigms, such as using chord music, or calling names to the music background, tended to have better outcomes [74]. And the P300 latency, which may represent an objective index of higher-order processing integration, could predict recovery of consciousness from VS/UWS to MCS [75]. However, absence of the P300 does not exclude a possible recovery of patients.

N400 is absent in the majority of DOC patients [76]. It does not reliably distinguish VS/UWS from MCS (in three levels: listening, cognition and speech) [77]. Both MCS and VS/UWS who have a preserved N400 show increased N400 peaks and amplitudes in the middle of the forehead during inconsistent speech stimulation, but the latency of N400 in VS/UWS is longer than that in MCS [78]. The relationship of N400 with the outcome of DOC patients is noticeable: In a clinical follow-up of 53 VS/UWS and 39 MCS, patients with preserved N400 had a highly significant relationship with the recovery of their communication ability (in MCS: sensitivity = 40%, specificity = 100%; in VS/UWS: sensitivity = 60%, specificity = 97%) [79]. The highest probability of recovery (97%) is observed for MCS with both detectable N400 and P300. In contrast, the lowest recovery chance (around 10%) is found for VS/UWS with neither a N400 nor a P300 [80]. Another study confirmed that presence of N400 is a predictive marker of communication recovery with sensitivity of 50% and specificity of 90% [81].

Moreover, some other cortical responses could also be evoked by auditory stimulation in some DOC patients, including mismatch negativity (MMN) [13, 67], late positive components [81] and lateralized readiness potential [82]. Late positive components and lateralized readiness potential were demonstrated as only existing in some MCS but not VS/UWS, which may be valuable in DOC diagnosis.

#### Visual evoked potentials

The flash visual evoked potential (VEP) is generated when subjects observe moving balls or objects. Compared with conscious states, the VEP amplitude in DOC patients is smaller and latency is longer. Initial VEP delays have important values in predicting the long-term prognosis (up to 3 years) [83]. However, as flash VEP requires active cooperation from patients, it is almost always difficult to be properly conducted in DOC patients. Some researchers use a novel approach to address this problem by employing an associative stimulation protocol combining transcranial magnetic stimulation (TMS) with visual stimulation through transorbital alternating current stimulation. This can serve to evaluate the visuomotor integration (VMI) and visual P300 patterns in DOC patients. MCS patients exhibit preserved patterns of VMI and P300, whereas nearly all VS/UWS patients did not show significant VMI [84].

### Brain–computer interfaces

Based on an external stimulus, a brain-computer interface (BCI) system directly detects brain responses rather than being limited to the subjective observation of patients and, therefore, increases objectivity and accuracy of conscious expression [85] (Table 5). Thus, BCI are used to assist clinical evaluation, especially CRS-R assessment, to obtain more accurate diagnoses. For example, for the auditory scare item in CRS-R, background noise was taken as the standard stimulus and clapping as the new stimulus. Traditionally, an assessor gives scores based on patients’ behavioral responses. However, by comparing the results of CRS-R and BCI, it was found that some patients cannot respond to the stimulus behaviorally but respond using an auditory BCI system (significant response waveforms following a loud clapping sound) [86, 87]. Similarly, in a visual BCI study some patients show significant evoked waveforms following commands of visual fixation, but fail to get a visual fixation score in CRS-R [88]. In addition, the visual BCI system proved to have better visual tracking ability than behavioral assessment in tasks of following a moving picture or identifying pictures of specific individuals [89, 90]. When using more complex cognitive tasks, BCI is sensitive to the patients’ covert consciousness expression. BCI using happy/sad emotional images shows that some DOC patients with insufficient behavioral response had significant emotional cognitive processing in the BCI system [91]. Moreover, some patients whose consciousness was detected by BCI but not behavioral assessment later have improved communication scores in their CRS-R [92]. Moreover, a gaze-independent BCI system combining auditory and visual inputs outperforms unimodal auditory-only and visual-only BCI systems. Multiple event-related potential (ERP) components, including the P300, N400 and late positive complex, could be observed only using the combined audio-visual BCI system [93]. Most patients, who respond in the audio-visual system, i.e., achieve statistically significant BCI accuracy, regain consciousness 3 months later [94].

In addition to the auditory and visual pathways, the somatosensory pathway shows important value in the DOC-BCI approach. BCI using vibratory tactile stimulation successfully identified patients without behavioral command-following but ‘covert command-following’ in the BCI system [95]. The patient, who responded in BCI showed a higher cerebral glucose metabolism within the language network indicative of MCS compared to other patients without responses in BCI [96]. The somatosensory discrimination BCI system also identified four VS/UWS patients who had clear task-following. The efficacy of somatosensory discrimination strongly correlates with the 6-month outcome [97]. This highlights the values of a somatosensory pathway-based BCI system in detecting ‘hidden command-following’ in patients with lack of behavioral response. It is also promising as an auxiliary method for predicting the clinical outcome of DOC patients. When using motor imagery but not sensory stimulation, some MCS patients show capacity to operate a simple BCI-based communication system, even without any detectable volitional control of movement [98]. Three of 16 VS/UWS patients had repeatedly and reliably appropriate EEG responses to commands in a motor-imagery-based BCI [99]. In addition, most patients with cognitive-motor dissociation show evidence of following different motor imagery commands, which indicates the possibility of re-establishing communication by imagery-based BCI for such patients [100].

### Electroencephalogram reactivity

EEG reactivity refers to changes of the brain electrical rhythm (frequency of EEG becoming faster or slower) or amplitude (increase or decrease) in response to external stimulation [101]. In DOC patients, EEG reactivity is more likely to occur in patients with a higher CRS-R [102]. EEG reactivity to intermittent photic stimulation and auditory stimulation is significantly higher in MCS than in VS/UWS. The preserved EEG reactivity evoked by eye opening/closing, auditory stimulation and intermittent photic stimulation has a high diagnostic specificity for the identification of patients with MCS vs. VS/UWS, while EEG reactivity to pain and tactile stimuli does not differentiate between MCS and VS/UWS [103].

### Transcranial magnetic stimulation-electroencephalogram

Since transcranial magnetic stimulation-electroencephalography (TMS-EEG) does not rely on the integrity of motor pathways (in contrast to recording TMS responses by electromyography from muscle) and does not require any active participation from the patient, TMS-EEG is now considered one of the most promising techniques in the diagnosis of DOC patients (Table 6).

The first TMS-EEG work in classifying DOC patients and tracking consciousness recovery was conducted in 2012 [104]. Similar to the findings from sleep and anesthesia research [105, 106], a breakdown of effective connectivity, i.e., a strongly reduced propagation of the initial EEG response at the site of stimulation throughout the brain, was also found in DOC patients. Moreover, the TMS evoked EEG potentials (TEPs) are significantly different between MCS and VS/UWS. MCS exhibits low-amplitude but high-frequency TEPs that propagate widely to distant cortical regions. In contrast, VS/UWS shows TEP waveforms similar to those observed in NREM sleep and deep anesthesia in HC, i.e. high-amplitude local EEG responses of low waveform complexity that do not propagate [104]. Another study confirmed that the TMS evoked cortical reactivity and connectivity are largely preserved in most MCS patients but not in VS/UWS patients [107]. These findings corroborate the notion that MCS patients retain extensive cortico-cortical connections for communication, which are seriously suppressed in VS/UWS patients. One proposition is that cortical circuits fall into silence and then further impair local causal interactions and prevent the build-up of global complexity in VS/UWS patients. Such OFF periods would be similar to those detected in sleep in healthy subjects [108]. This concept was further confirmed by TEP measurements along with modification by transcranial direct current stimulation (tDCS) in DOC patients [109]. More cerebral regions could be re-excited by tDCS (increased temporal and spatial distributions of TEPs after tDCS than before) in MCS than VS/UWS, suggesting global breakdown and silent cortexes in VS/UWS patients.

**Fig. 2:**
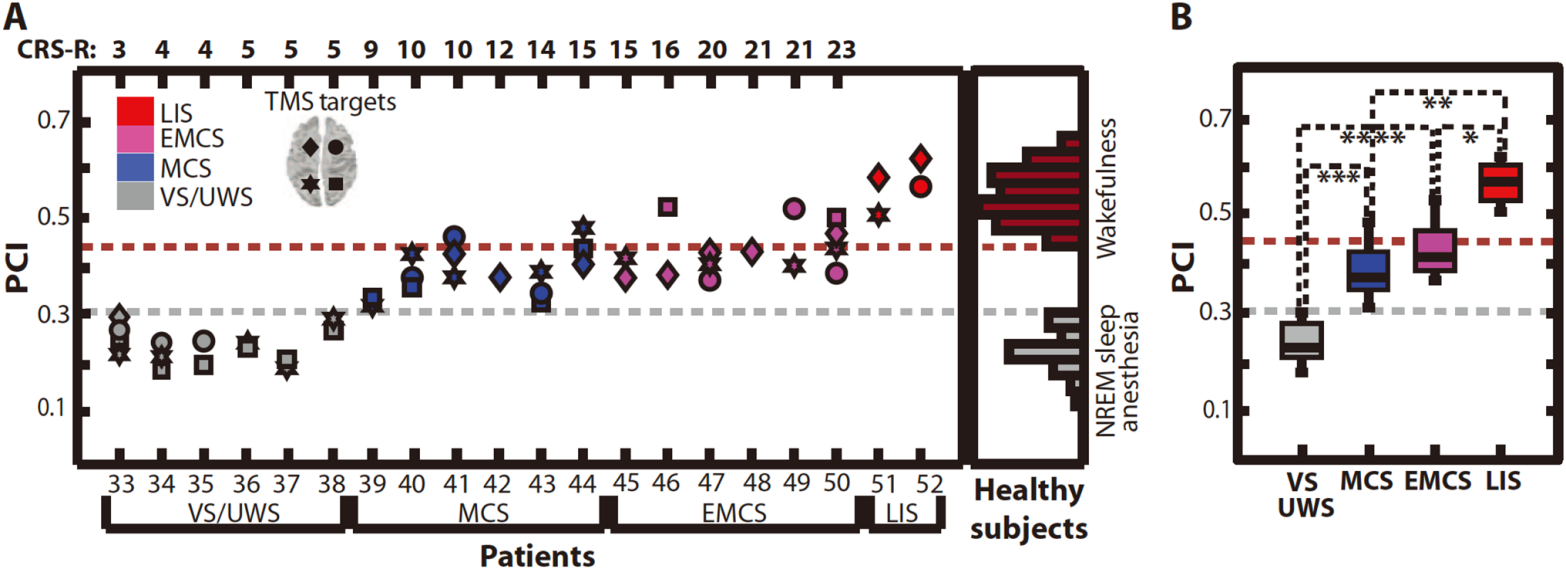
Perturbational complexity index (PCI) discriminates the level of consciousness in brain-injured patients. **a** PCI values for 48 TMS sessions collected from 20 severely brain-injured patients (TMS was targeted to both left and right Brodmann areas 8 and 7, as indicated at top left). Right: distribution of PCI values from healthy individuals. **b** Box plots for PCI in brain-injured patients with the statistical significance between pairs of conditions (Linear mixed-effects models: *P = 0.002, **P = 0.0001, ***P = 2 × 10^−5^, ****P = 8 × 10^−7^). Gray and red dashed lines in (**a**) and (**b**) represent the maximum complexity observed during unconsciousness (PCI = 0.31) and the minimum complexity observed during alert wakefulness (PCI = 0.44) in healthy subjects, respectively (with permission, from Casali et al. [110] Sci Transl Med 5:198ra105)

The perturbation complex index (PCI) was proposed to quantify the temporal-spatial complexity of TEPs (Fig. 2) [110]. PCI reflects the ability of integration information of cortex, which is considered as one of the key neurophysiological bases of human consciousness [111]. PCI could effectively distinguish the consciousness levels as follows: values under 0.31 as VS/UWS, 0.32–0.49 as MCS, 0.37–0.52 as emerged from MCS, 0.51–0.62 as LIS and 0.44–0.67 as awake [110]. A large sample study (43 VS/UWS and 38 MCS) has measured multiple times PCI from each patient [112]. Finally, the threshold of maximum PCI reached 86.4% accuracy of identifying MCS and VS/UWS (total sensitivity = 80%, specificity = 94.4%). In addition, Bodart et al. [113] combined PCI, CRS-R and FDG-PET, and demonstrated congruent data for PCI and PET in 22 of the 24 DOC patients: preserved metabolism in the fronto-parietal network corresponds to PCI above 0.31. Moreover, four patients classified as VS/UWS by CRS-R but high PCI eventually showed good clinical outcome. This is consistent with other findings that most VS/UWS patients with PCI > 0.31 recover to MCS eventually (accuracy = 73%, sensitivity = 54.5%, specificity = 84.2%) [112]. Furthermore, along with a progressive clinical improvement of a MCS patient in a repetitive TMS treatment, the PCI also tended to gradually increase [114]. In summary, PCI is a diagnostic, monitoring and prognostic biomarker of high potential value in the management of DOC patients.

## Discussion and conclusion

Although we still lack full knowledge about the electrophysiological foundations of human consciousness, numerous EEG studies were instrumental in developing current practice in handling DOC patients. Many EEG measures improve classification accuracy of DOC. Especially in patients who are not able to express conscious responses in behavioral assessment, bedside EEG measurements reduce rate of misdiagnosis [97, 99], and even reestablish the capability of DOC patients of communicating with the outside world [87]. At the same time, EEG measures inform physicians about neural responses to specific treatment. EEG offers the opportunity of testing the efficacy of different treatment approaches, as there is still no distinctly effective treatment strategy for DOC patients [115].

EEG studies have important implications for medicolegal decision making in DOC patients. However, the variety and complexity of EEG measures and analyses create obstacles for becoming a practical and widely applicable tool. Resting-state EEG captures spontaneous activities of neuronal assembles. However, these activities have properties of non-linear dynamics, transitions and high complexity. In addition, the signal analysis decodes information from different dimensions based on EEG amplitude, frequency, phase, time frequency, phase amplitude coupling, etc. Combined with external stimulation, EEG information becomes even more complicated. Different types of stimulation evoke responses from different neural pathways and circuits. Moreover, responses at different latencies carry different information. These complicated multitude of EEG characteristics threatens to become a burden rather than a help in the management of DOC patients. A new question need to be answered: Which one of the numerous EEG measures is the best? Some comparative studies were conducted using relatively large samples of DOC patients to test the utility of various EEG measures in diagnostic accuracy or prediction of outcome [13, 30]. Such studies will help to sort out the most valuable consciousness-related EEG characteristics and facilitate their application in DOC clinics.

When using EEG for diagnostic assessment, the lack of a ‘gold standard’ for detection of conscious awareness is the most prominent limitation. EEG classification accuracy is extremely affected by the clinical diagnosis. Multiple studies conducted classification statistics based on diagnosis from behavioral assessment, such as CRS-R. However, a significant fraction of patients is misdiagnosed based on behavioral assessments, which confounds the performance and interpretation of the EEG measures. Combining EEG with other methods, such as fluoro-2-deoxy-d-glucose-positron emission tomography [52] or functional magnetic resonance imaging [100] has highlighted the potentially excellent diagnostic performance of the EEG measures. Especially, with assistance of BCI systems, it was demonstrated that many DOC patients are misdiagnosed by behavioral assessment [99]. Therefore, inconsistency between EEG classification and behavioral diagnosis should not lead to the conclusion of diagnostic inaccuracy of the EEG but rather to seek for additional diagnostic information through other neuroimaging techniques.

Current studies often show differences of EEG measures at the group level (e.g., MCI vs. VS/UWS). The group statistics cannot be used for individual diagnosis. The recently developed TMS-EEG technique is currently considered the most promising diagnostic method at the individual level. The relationship of TME-EEG characteristics with human consciousness was established from sleep and anesthesia research, demonstrating that ranges of the perturbational complexity index reliably classify VS/UWS, MCS and healthy individuals [112]. Determination of objective threshold values is a critical step towards building an individual diagnostic system and its clinical application.

EEG measures show potential value in predicting DOC outcome. Presence of sleep-related EEG components, such as sleep spindles, or the N400 in event-related potential recordings usually indicate good recovery [79, 116]. However, it should be noted is that prognostic studies in DOC patients were confounded by the medical treatment strategies, nursing and complication management of the patients. For example, if MCS patients were misdiagnosed as VS/UWS, they were more likely to be transferred to nursing homes or other facilities unable to provide best standards of medical care. Therefore, the prognostic values of EEG measures in DOC patients still await controlled, large-scale multicentre validation studies.

Finally, EEG studies in DOC patients are difficult to perform. Heterogeneity in the etiology of DOC likely imposes the problem of a lack of unifying EEG characteristics. Simple practical issues, such as motor restlessness, a characteristic feature of many MCS patients, makes EEG measurement difficult or impossible. Moreover, sequelae caused by brain injury, such as epilepsy or paroxysmal sympathetic hyperactivity, may contaminate the EEG signal. In addition, EEG characteristics, which might show diagnostic or prognostic utility in some studies will likely not always perform well in real-world clinical applications due to limited sample sizes and specific patient characteristics in the studies. Despite these limitations, the current review still identified some specific EEG characteristics with consistently reliable performance across studies in identifying the consciousness level and predicting outcome of DOC patients, such as resting-state alpha power in MCS vs. VS/UWS classification [11, 30], N400 in MCS recovery prediction [79, 80], and PCI (from TMS-EEG) in both individual diagnosis and prognosis [110, 112]. Moreover, machine learning trained by comprehensive EEG characteristics, including indexes derived from different information dimensions, could achieve high levels of accuracy in diagnosis and prognosis of DOC patients [30, 52].

Despite all difficulty and immaturity, this review has provided manifold evidence that the EEG can assess the dysfunctional brain networks of DOC patients, resulting in relevant improvement in diagnostic accuracy, and opening up the opportunity of monitoring treatment effects and predicting long-term outcome. This will help the professional handling of DOC patients.
